# The lncRNA H19/miR-766-3p/S1PR3 Axis Contributes to the Hyperproliferation of Keratinocytes and Skin Inflammation in Psoriasis via the AKT/mTOR Pathway

**DOI:** 10.1155/2021/9991175

**Published:** 2021-12-28

**Authors:** Yuexi He, Xiran Yin, Jianjun Yan, Xue Li, Qing Sun

**Affiliations:** ^1^Department of Dermatology, Qilu Hospital, Cheeloo College of Medicine, Shandong University, Jinan, Shandong 250012, China; ^2^Department of Dermatology, Yantai Yuhuangding Hospital of Qingdao University, Yantai, Shandong 264000, China

## Abstract

**Background:**

The pathogenesis of long noncoding RNAs (lncRNAs) and microRNAs (miRNAs) are well studied in psoriasis. However, little is known about how specific lncRNAs and miRNAs affect the mechanism of psoriasis development and which pathways are involved.

**Objectives:**

To explore the role of the lncRNA H19/miR-766-3p/S1PR3 axis in psoriasis.

**Methods:**

miRNA and lncRNA microarrays were performed using IL-22-induced HaCaT cells and psoriatic lesions, respectively. Fluorescence *in situ* hybridization and quantitative reverse-transcriptase polymerase chain reaction were used to detect the expression of miR-766-3p and lncRNA H19. Luciferase reporter assays were used to identify miR-766-3p/lncRNA H19 and miR-766-3p/S1PR3 combinations. CCK-8 and ELISA were performed to evaluate the proliferation of keratinocytes and the secretion of pro-inflammatory cytokines. Western blot analysis was used to detect the expression of S1PR3 and its downstream effector proteins.

**Results:**

MiR-766-3p was upregulated in both HaCaT cells treated with the psoriasis-related cytokine pool (IL-17A, IL-22, IL-1 alpha, oncostatin M, and TNF-alpha) and tissues. Overexpression of miR-766-3p promoted keratinocyte proliferation and IL-17A and IL-22 secretion. LncRNA H19 and S1PR3 were demonstrably combined with miR-766-3p by luciferase reporter assay. lncRNA H19 repressed proliferation and inflammation, which were reduced by the miR-766-3p. AKT/mTOR pathway effected proliferation and inflammation by the lncRNA H19/miR-766-3p/S1PR3 axis.

**Conclusions:**

We established that downregulation of lncRNA H19 promoted the proliferation of keratinocytes and skin inflammation by up-regulating miR-766-3p expression levels and inhibiting activation of S1PR3 through the AKT/mTOR pathway in psoriasis.

## 1. Introduction

Psoriasis is a chronic inflammatory, immune-mediated disease that manifests in the skin, joints or other systematics, especially cardiovascular system. It is associated with both physical and psychological burdens [1]. It is characterized by erythaematous scaly patches or plaques [2]. Psoriatic skin lesions are the result of an intricate interplay between the innate and adaptive components of the immune system [1, 3]. Recent studies have shown that epigenetics is an important component of psoriasis aetiology in general [2–4]. However, the exact underlying mechanisms regulating immunological dysfunction have not been completely elucidated.

Long noncoding RNAs (lncRNAs) are a group of RNAs that are longer than 200 nucleotides and have no ability to encode proteins [5]. However, lncRNAs play an important role in the control of cell fates during development and cause some human disorders by facilitating chromosomal deletions and translocations [6]. LncRNA H19 has been widely investigated in many diverse disorders. For instance, lncRNA H19 plays crucial roles in several inflammatory diseases, such as cardiovascular disease, atherosclerosis, osteoarthritis, and collagen-induced arthritis [7–10]. Additionally, specific lncRNAs, such as MSX2P1, MIR31HG, and PRINS, have been shown to participate in the regulation of psoriasis by influencing the hyperproliferation of keratinocytes and their and inflammatory capabilities [11–13]. Our previous study reported the differential expression of lncRNAs in biopsies obtained from psoriasis patients and healthy volunteers using a microarray [14]. Interestingly, lncRNA H19 was found to have a downregulation (0.248121-fold) in the study. However, whether lncRNA H19 influences psoriasis remains unknown.

microRNAs (miRNAs), noncoding small RNAs (~22 nucleotides), were discovered to play an important role in many disorders, regulating diverse biological processes such as development and cell apoptosis, proliferation and differentiation [15]. Many miRNAs were found to have effects on psoriasis. For example, the downregulation of miR-145-5p expression contributes to hyperproliferation and inflammation in psoriasis [16]. In contrast, miR-744-3p promoted keratinocyte proliferation while inhibiting their differentiation [17]. Recently, convincing evidence has indicated that miR-766-3p can induce or inhibit multiple human cancers and suppress inflammatory responses [18–20]. The role of miR-766–3p in psoriasis, however, remains elusive.

In the study, we demonstrated that lncRNA H19 was markedly down-regulated in tissue samples and HaCaT cells treated with IL-17A, IL-22, IL-1 alpha, oncostatin M, and TNF-alpha. LncRNA H19 might serve as a sponge for miR-766-3p to up-regulate S1PR3 levels and regulates the proliferation of keratinocytes and skin inflammation by AKT/mTOR pathway in psoriasis. Therefore, our findings may provide novel evidences for the clinical therapeutic strategies for psoriasis treatment.

## 2. Materials and Methods

### 2.1. Patients and Sample Collection

Six specimens were taken from patients diagnosed with psoriasis vulgaris at the Qilu Hospital of Shandong University who had received no systemic treatments, phototherapy or externally used drugs for at least 3 months before the skin biopsies. Healthy skin from surgical operations was used as control. The study was approved by the ethics committee of Shandong University, China, and all patients provided written informed consent.

### 2.2. Cell Culture and Treatment with M5

The human keratinocyte cell line (HaCaT) from the China Center for Type Culture Collection (Wuhan, China) was cultured in Dulbecco's modified Eagle's Medium (DMEM) (Gibco, U.S.A.) supplemented with 10% foetal bovine serum (FBS) (Sangon Biotech, China), 100 *μ*g/ml streptomycin and 100 U/ml penicillin. Cells were seeded for subsequent experiments in 6-, 24-, or 96-well plates with M5 (IL-17A, IL-22, IL-1 alpha, oncostatin M, and TNF-alpha, 10 ng/mL).

### 2.3. Transfection

Cells were transfected with miR-766-3p mimics or an miR-766-3p mimic negative control (NC); an miR-766-3p inhibitor or an miR-766-3p inhibitor negative control (NC) (Ribobio, China); or the PGMLV-H19 plasmid or negative control (NC) (Genomeditech, China) using lipofectamine 2000 (Invitrogen, U.S.A.); or AKT inhibitor MK-2206 (Selleck, U.S.A.) according to the manufacturer's instructions. Cells were collected for further treatment or analysis at different time points after transfection.

### 2.4. CCK-8 Assay

Cell proliferation rates were measured using CCK-8 assays (Dojindo, Japan) at 24, 48 and 72 h after being transfected as described above. The optical density was measured at 450 nm by a Synergy H1 Microplate Reader (BioTek, U.S.A.).

### 2.5. Fluorescence *in Situ* Hybridization Analysis

Tissue sections embedded in paraffin were hybridized with the following digoxigenin-labelled probes: miR-766-3p (5'-DIG-GCTGAGGCTGTGGGGCTGGAGT-DIG-3') and lncRNA H19 (5'-DIG-GCTGT TCCGA TGGTG TCTTT GATGT TGGGC TGATG-DIG-3'). DAPI was used for cell nuclei staining. (Servicebio, China).

### 2.6. Luciferase Reporter Assay

The recombinant pmirGLO-H19-MUT (mutant) and pmirGLO-H19-WT (wild-type) plasmids and the pmirGLO-S1PR3-MUT (mutant) and pmirGLO-S1PR3-WT (wild-type) plasmids were purchased from RiboBio (Guangzhou, China). Cells were cotransfected with a miR-766-3p mimic or negative control (50 nM) and pmirGLO-H19-WT or pmirGLO-H19-MUT; pmirGLO-S1PR3-MUT and pmirGLO-S1PR3-WT (250 ng per well) with Lipofectamine 2000. The luciferase level was detected using the Dual-Luciferase Reporter Assay System (Promega, U.S.A.) after 48 h.

### 2.7. Quantitative Reverse Transcriptase Polymerase Chain Reaction (qRT-PCR)

Total RNA was extracted from the samples described above using TRIzol reagent (Invitrogen, U.S.A.). The miRNA and mRNA expression were detected according to the manual of the All-in-One miRNA qRT-PCR Detection System (GeneCopoeia, U.S.A.) and the PrimeScript RT reagent kit with gDNA Eraser and TB Green Premix Ex Taq II (Takara, Japan). The expression levels were normalized to U6 or GAPDH. The sequences of the miR-766-3p and U6 primers were designed by GeneCopoeia (HmiRP0794 and HmiRQP9001). LncRNA and mRNA sequences used for qPCR are shown in Table 1. We conducted independently repeated experiments at least three times and determined expression by the 2-*ΔΔ*Ct formula.

### 2.8. Western Blotting

Western blotting was performed as previously described [21, 22]. The following primary antibodies were used in this study: Rabbit mAb GAPDH (1 : 1000, #5174), Phospho-Akt (1: 2000, #4060), Akt (1 : 1000, #4691), Phosoho-mTOR (1 : 1000, #5536), mTOR (1 : 1000, #2983) from Cell Signalling Technologies, Beverly, MA, U.S.A, and S1PR3 (1 : 10000, ab108370) from Abcam, Cambridge, U.K.

### 2.9. Elisa

Cell supernatant was separated, and stored at −80°C until analysis. The level of IL-17A and IL-22 were measured by enzyme-linked immunosorbent assay (ELISA) (Elabscience, China) kits according to the manufacturer's instructions. The optical density was measured at 450 nm by a Synergy H1 Microplate Reader (BioTek, U.S.A.).

### 2.10. Immunohistochemistry

Immunohistochemistry (IHC) was performed as previously described [21, 22]. The primary antibody used in this study was S1PR3 (1 : 10000, ab108370) from Abcam, Cambridge, U.K.

### 2.11. Statistical Analysis

Data from at least three independent experiments were presented as the mean +/- standard deviation (SD). Comparisons were performed using Student's t-test, and p <0.05 was considered statistically significant.

## 3. Results

### 3.1. microRNA Microarray Validation and Target Prediction

**Figure 1:**
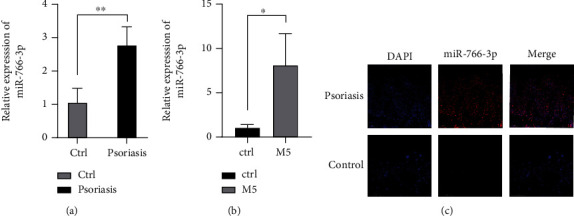
microRNA microarray validation and target prediction. (a) Expression level of miR-766-3p in psoriasis tissue (n =3). (b) Expression level of miR-766-3p in M5-induced HaCaT cells. (c) Fluorescence *in situ* hybridization showed the localization of miR-766-3p in psoriasis tissue. Scale bar: 50 *μ*m. Data were shown as the mean ± SD, ∗P <0.05, ∗∗ P<0.01. All the experiments were repeated at least three times.

According to the results of the microarrays in our previous studies [21, 22], the expression of miR-766-3p was increased 2.548-fold, and we verified its expression in psoriasis tissues and M5-stimulated HaCaT cells by qRT-PCR (Figures 1(a) and 1(b)]). We found that the expression of miR-766-3p was significantly upregulated in psoriasis tissue samples versus normal control tissues. Similarly, miR-766-3p expression in HaCaT cells stimulated with M5 was significantly higher than that in control. We performed fluorescence *in situ* hybridization (FISH) to detect the expression of miR-766-3p in the epidermis of psoriasis tissues, and found that miR-766-3p expression was upregulated in psoriatic tissues versus control tissues (Figure 1(c)).

### 3.2. Proliferative and Inflammatory Effects of miR-766-3p on HaCaT Cells

**Figure 2:**
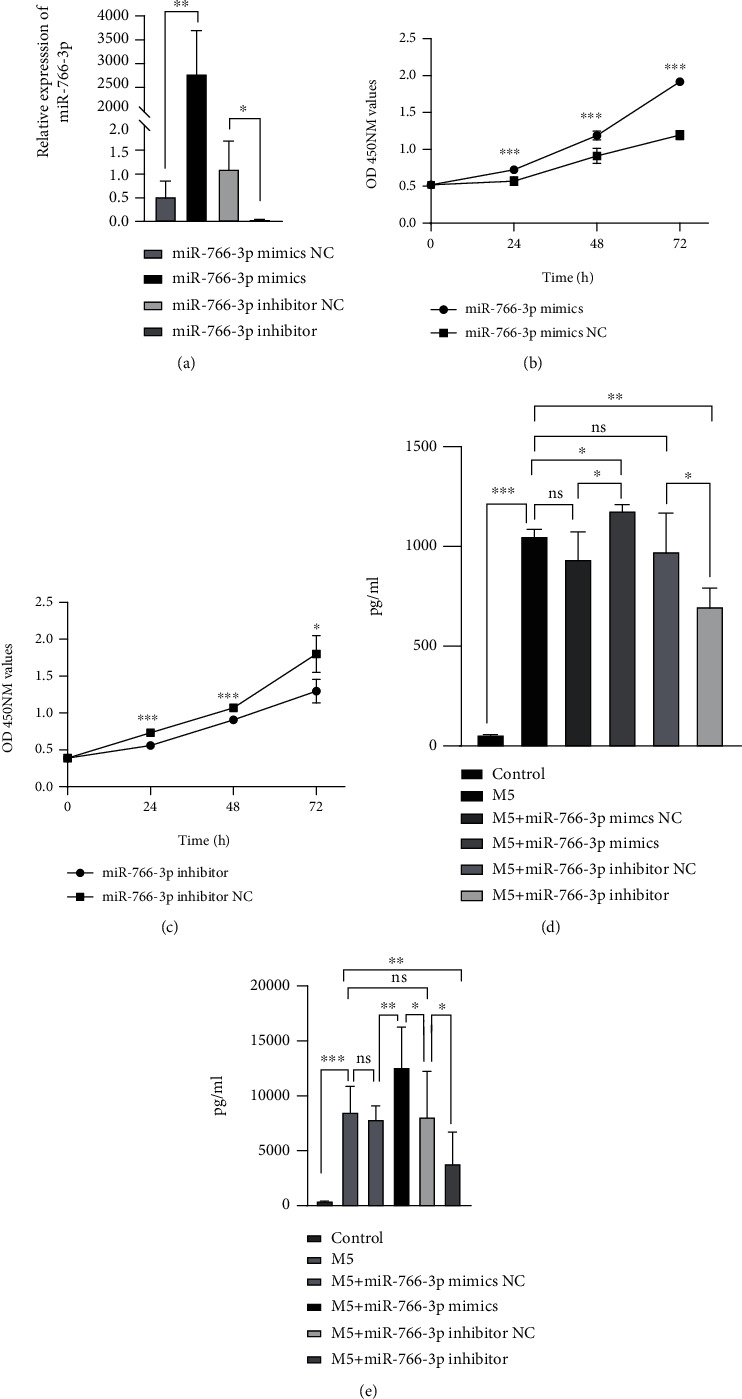
Proliferative and inflammatory effects of miR-766-3p on HaCaT cells. (a-c) HaCaT cells were transfected with miR-766-3p mimics/NC and inhibitor/NC; miR-766-3p expressions were detected with qRT-PCR (a); proliferative effects were assessed by CCK-8 (b-c). (d-e) HaCaT cells were treated with M5; ELISA showed the expression level of IL-17A (d) and IL-22 (e). Data were shown as the mean ± SD, ∗P <0.05, ∗∗ P<0.01, ∗∗∗P<0.001. All the experiments were repeated at least three times.

To understand the potential impact of miR-766-3p on the behaviour of M5-induced HaCaT cells, we transfected miR-766-3p mimics, inhibitors or negative control miRs into HaCaT cells. The data obtained by qRT-PCR showed the efficiency of miR-766-3p mimic and inhibitor expression in the transfected cells (Figure 2(a)). The result of the CCK-8 proliferation assay showed that overexpression of miR-766-3p significantly promoted the proliferation of HaCaT cells (Figure 2(b)). In contrast, downregulation of miR-766-3p expression inhibited cell proliferation (Figure 2(c)). These data demonstrated that miR-766-3p might play an important role in the abnormal proliferation of keratinocytes in psoriasis. The secretion of inflammatory factors by M5-induced HaCaT cells was assessed by ELISA. The results indicated that miR-766-3p mimics promoted the secretion of IL-17A (Figure 2(d)) and IL-22(Figure 2(e)) after M5 treatment. Conversely, miR-766-3p inhibited the secretion of these factors.

### 3.3. MiR-766-3p Negatively Regulates lncRNA H19

**Figure 3:**
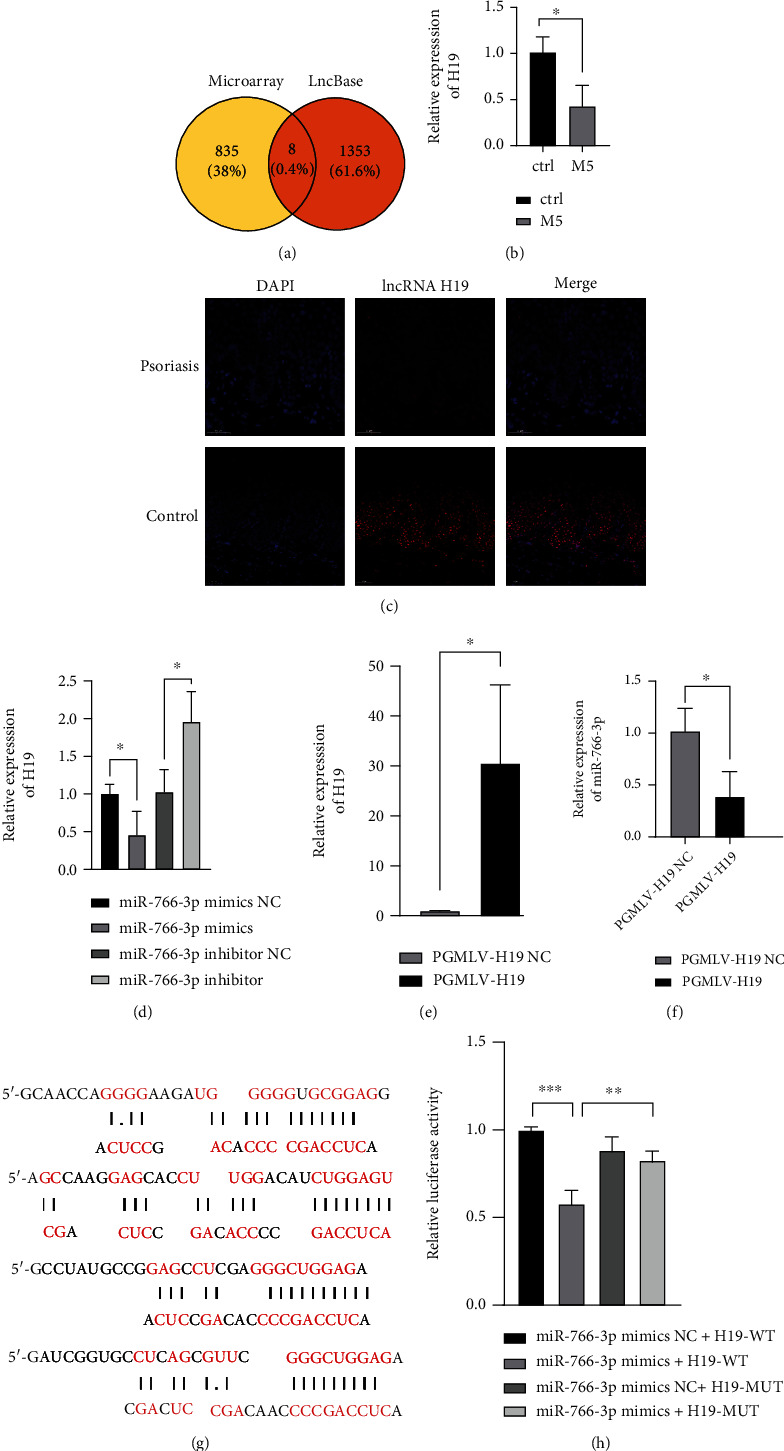
MiR-766-3p negatively regulates lncRNA H19. (a) Prediction of miR-766-3p by LncBase v.2 and microarray. (b) Expression level of lncRNA H19 in M5-induced HaCaT cell. (c) Fluorescence in situ hybridization showed the localization of lncRNA H19 in psoriasis tissue. Scale bar: 50 *μ*m. (d) Expression level of lncRNA H19 in HaCaT cells by qRT-PCR. (e-f) HaCaT cells were transfected with PGMLV-H19/NC; lncRNA H19 expressions were detected with qRT-PCR (e); miR-766-3p expressions were detected with qRT-PCR (f). (g) The partial binding sites between miR-766-3p and lncRNA H19 are shown. (h) Luciferase activity was detected in HEK293 cells cotransfected with miR-766-3p mimics/NC and WT and MUT H19. Data were shown as the mean ± SD, ∗P <0.05, ∗∗∗P<0.001. All the experiments were repeated at least three times.

To investigate the mechanism of miR-766-3p in psoriasis, we predicted its potential target lncRNA using LncBase v.2 and the microarray we analysed previously (Figure 3(a)) [14]. MiRNAs usually work through a ceRNA network, which functions as a sponge [23]. There were eight lncRNAs identified as potential binding targets of miR-766-3p that are downregulated in psoriasis. We then detected the expression of lncRNA H19 which demonstrated a 0.248121-fold decrease in expression in psoriasis. We found that lncRNA H19 expression was decreased in M5-induced HaCaT cells by qRT-PCR (Figure 3(b)). The FISH results showed that the expression of lncRNA H19 was downregulated in psoriatic skin (Figure 3(c)). The qRT-PCR results indicated that miR-766-3p overexpression considerably reduced lncRNA H19 expression and downregulation of miR-766-3p expression increased the expression of lncRNA H19 (Figure 3(d)). The overexpression of lncRNA H19 via the PGMLV-H19 plasmid was successful (Figure 3(e)) and correspondingly reduced the expression of miR-766-3p (Figure 3(f)). The potential binding sites between lncRNA H19 and miR-766-3p are shown in Figure 3(g) and a luciferase reporter assay was performed to explore the relationship between miR-766-3p and lncRNA H19. Our results showed that the relative activity of miR-766-3p was decreased in the wt lncRNA H19 group and showed no significant difference in the mut lncRNA H19 group (Figure 3(h)). These results demonstrated that lncRNA H19 can directly combine with miR-766-3p.

### 3.4. MiR-766-3P Targeted S1PR3

**Figure 4:**
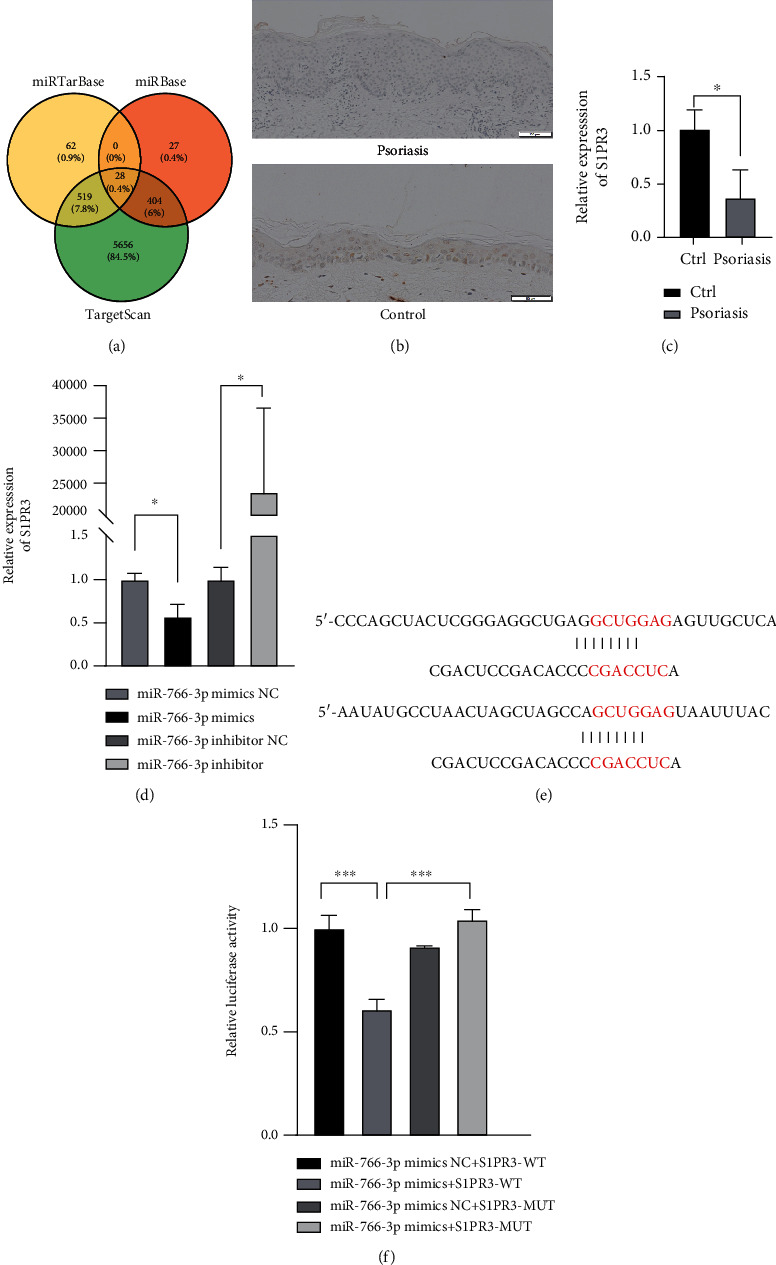
MiR-766-3P targeted S1PR3. (a) Prediction of miR-766-3p by TargetScan, miRBase and miRTarBase. (b) Immunohistochemistry of S1PR3 in normal and psoriasis tissues. (c) Expression level of S1PR3 in psoriasis tissues by qRT-PCR (n =3). (d) Expression level of S1PR3 in miR-766-3p mimics/NC- or inhibitor/NC-induced HaCaT cells by qRT-PCR. (e) The partial binding sites between miR-766-3p and S1PR3 are shown. (f) Luciferase activity was detected in HEK293 cells cotransfected with miR-766-3p mimics/NC and WT and MUT S1PR3. Data were shown as the mean ± SD, ∗P <0.05, ∗∗∗P<0.001. All the experiments were repeated at least three times.

Next, TargetScan, miRBase and miRTarBase were applied to predict the potential target genes of miR-766-3p (Figure 4(a)). We found 28 potential target genes and selected S1PR3, which is associated with the Ras/pERK and PI3K/AKT pathways [24, 25] reported in psoriasis and plays role in pro-inflammatory cytokine regulation [26]. We found that expression of S1PR3 was downregulated in psoriasis tissue (Figures 4(b) and 4(c)). The qRT-PCR results suggested that the expression of S1PR3 was regulated by miR-766-3p; meanwhile, miR-766-3p inhibitors increased S1PR3 expression (Figure 4(d)). The binding site of S1PR3 and miR-766-3p was determined by a dual-luciferase reporter assay (Figure 4(e)). The wt S1PR3 luciferase vector had miR-766-3p binding sites, while the mut S1PR3 luciferase vector lacked miR-766-3p binding sites (Figure 4(f)).

### 3.5. The lncRNA H19/miR-766-3p/S1PR3 Axis Affects Cell Proliferation and Inflammation in Psoriasis via the AKT/mTOR Pathway

**Figure 5:**
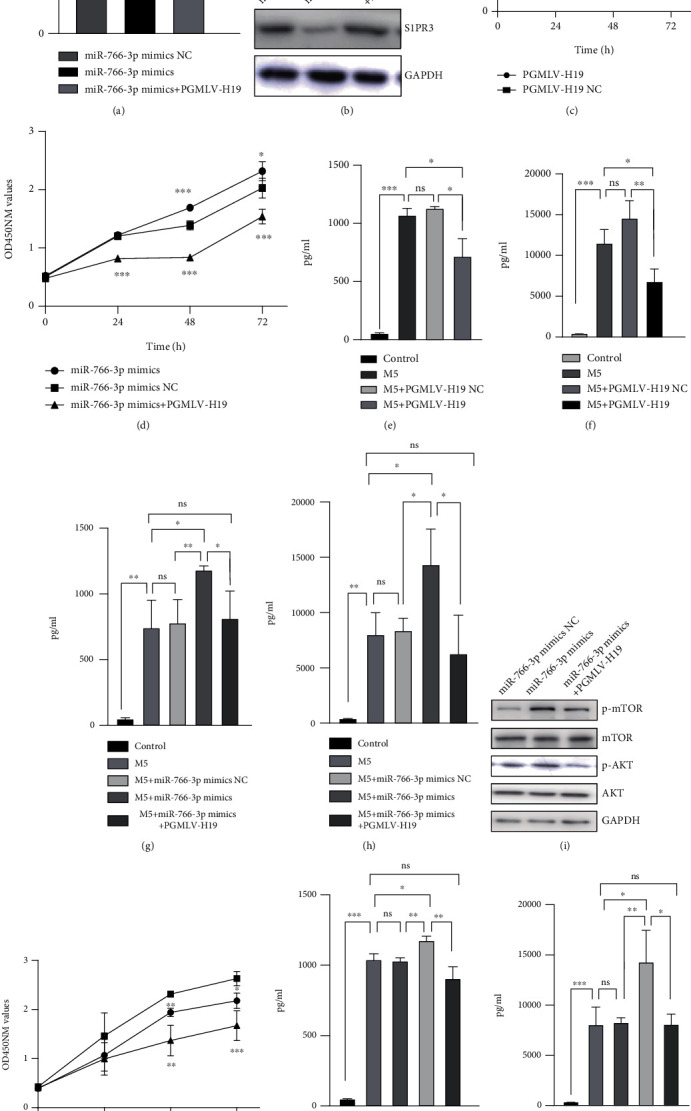
The lncRNA H19/miR-766-3p/S1PR3 axis affects cell proliferation and inflammation in psoriasis via the AKT/mTOR pathway. (a-b) Cotransfection of PGMLV-H19 plasmid with miR-766-3p mimics in M5-induced HaCaT cells; expression level of S1PR3 by qRT-PCR (a); and western blot (b). (c) Proliferative effects of lncRNA H19 were assessed by CCK-8. (d) Proliferative effects of cotransfection of PGMLV-H19 plasmid with miR-766-3p mimics were assessed by CCK-8. (e-h) HaCaT cells were transfected with PGMLV-H19/NC (e-f) and cotransfection of PGMLV-H19 plasmid with miR-766-3p mimics (g-h); ELISA showed the expression level of IL-17A (e, g) and IL-22 (f, h). (i) Western blot showed the expression level of p-mTOR, mTOR, AKT, p-AKT in M5-induced HaCaT cells transfected with PGMLV-H19 plasmid and miR-766-3p mimics. (j-l) Cotransfection of miR-766-3p mimics with MK-2206; proliferative effects were assessed by CCK-8 (j); ELISA showed the expression level of IL-17A (k) and IL-22(l). Data were shown as the mean ± SD, ∗P <0.05, ∗∗P <0.01, ∗∗∗P<0.001. All the experiments were repeated at least three times.

To further demonstrate the relationship between lncRNA H19, miR-766-3p and S1PR3, rescue experiments were performed using M5-induced HaCaT cells. Cotransfection of the PGMLV-H19 plasmid with miR-766-3p mimics resulted in the upregulation of S1PR3 expression over that in cells transfected with the miR-766-3p mimics (Figures 5(a) and 5(b)). The CCK-8 assay showed that compared with the negative control, lncRNA H19 obstructed cell proliferation (Figure 5(c)). Cotransfection of lncRNA H19 and miR-766-3p negated the proliferative effect of lncRNA H19 (Figure 5(d)). The ELISA revealed that overexpression of lncRNA H19 decreased the levels of inflammatory factors IL-17A and IL-22 (Figures 5(e) and 5(f)). In addition, co-transfection with miR-766-3p reversed the effect of lncRNA H19 (Figures 5(g) and 5(h)). In our exploration of the role of lncRNA H19 and miR-766-3p in psoriasis, we found that the AKT/mTOR pathway was activated by overexpression of miR-766-3p and that lncRNA H19 lessened the expression of the pathway (Figure 5(i)). Furthermore, cotransfection of miR-766-3p mimics with AKT inhibitor MK-2206 resulted in proliferation (Figure 5(j)) and inflammation (Figures 5(k) and 5(l)) inhibition of HaCaT cells.

## 4. Discussion

Psoriasis is a chronic inflammatory, immune-mediated disease [1] which is induced by many pro-inflammatory cytokines, such as IL-17A, TNF-a, IL-22 and IL-23 [27]. IL-23/IL-17A axis plays a central role in the development of psoriasis. The combination of IL-17A, IL-22, IL-1 alpha, oncostatin M, and TNF-alpha (M5) inhibits the differentiation of keratinocytes and prolongs keratinocyte life [28]. Several studies have suggested that miRNAs might play key roles in psoriasis, including in proliferation regulation and cytokine secretion. In the future, miRNA may be identified as a biomarker and treatment target for psoriasis [29]. The AKT/mTOR pathway plays an important role in epidermal homeostasis control. AKT promotes cell proliferation and inhibits apoptosis. mTOR, downstream of AKT, facilitates cell proliferation, inhibits maturation [30] and regulates the release of pro-inflammatory mediators of keratinocytes [31]. In this study, we found an aggravated association between miR-766-3p expression and psoriasis. Overexpression of miR-766-3p was identified to facilitate the proliferation of keratinocytes and their inflammatory capabilities. LncRNA H19 and S1PR3 were the upstream and downstream targets of the miR-766-3p sponge. MiR-766-3p antagonized the negative regulation of proliferation and inflammation induced by lncRNA H19. The lncRNA H19/miR-766-3p/S1PR3 axis might act via the AKT/mTOR pathway.

Our miRNA microarray showed the expression of 20 different miRNAs were altered >2-fold including 15 upregulated and 5 downregulated miRNAs [22]. MiR-766-3p expression increased 2.548-fold in the microarray and is involved in many proliferative cancers and inflammatory disorders [18–20]. We conducted experiments to verify the expression and function of miR-766-3p in a psoriatic cell model. We found that miR-766-3p expression increased in paraffin-embedded psoriasis tissue by FISH and that miR-766-3p RNA levels were increased by qRT-PCR which verified the result of miRNA microarray. Next, we tested miR-766-3p expression in M5-induced HaCaT cells and found the same result. By a CCK-8 proliferation assay, we corroborated that miR-766-3p positively regulates the proliferation of HaCaT cells. Meanwhile, the inflammatory effect of miR-766-3p was substantiated by ELISA for IL-17A and IL-22. The result demonstrated that miR-766-3p facilitates IL-17A and IL-22 secretion.

LncRNA-miRNA-mRNA network may play important role in psoriasis and some of them are predicted or proved [32]. Furthermore, we wanted to discuss the ceRNA-mediated network of miR-766-3p which acts as a molecular sponge. We predicted the potential lncRNAs that combine with miR-766-3p and searched for lncRNAs that were also identified in the microarray on psoriasis tissue. Downregulation of lncRNA H19 in psoriasis tissues compared with normal tissues was observed in profiling studies [33]. We confirmed that the expression of lncRNA H19 was downregulated in psoriatic tissue and cells. Moreover, the positive and negative regulatory effects of miR-766-3p and LncRNA H19 were reciprocal. LncRNA H19 is one of the best-understood lncRNAs and plays a vital role in inflammatory diseases such as cardiovascular disease, atherosclerosis, and osteoarthritis, collagen-induced arthritis [7–10]. LncRNA H19 promotes cell proliferation in several disorders, such as pancreatic cancer, hepatocellular carcinoma and bladder cancer [34–36]. In contract, lncRNA H19 inhibits cell proliferation in pituitary tumours [37]. LncRNA H19 competes with coding Dsg1 for miR-130b-3p, thereby leading to increased Dsg1 expression, which promotes keratinocyte differentiation [38]. We found that overexpression of lncRNA H19 impeded cell proliferation and IL-17A and IL-22 secretion. We predicted that lncRNA H19 effects psoriasis by sponging miR-766-3p in proliferation and inflammation.

S1PR3 was predicted to be the target gene of miR-766-3p, which is associated with the psoriasis-related Ras/pERK and PI3K/AKT pathways [24, 25] and acts as a pro-inflammatory cytokine [26]. We confirmed that S1PR3 was downregulated in psoriasis tissues and M5-induced HaCaT cells. MiR-766-3p negatively regulated S1PR3. Subsequent functional studies disclosed the function of the lncRNA H19/miR-766-3p/S1PR3 axis. We found that miR-766-3p reversed the effect of overexpression of lncRNA H19 on S1PR3 expression. Meanwhile, the upregulation of miR-766-3p could rescue the proliferative and inflammatory effects exerted on keratinocytes by lncRNA H19 overexpression. The lncRNA H19/miR-766-3p/S1PR3 axis affected the activation of AKT/mTOR pathway and proliferation of keratinocytes and skin inflammation.

In conclusion, we have established that downregulation of lncRNA H19 promoted the proliferation of keratinocytes and skin inflammation by up-regulating miR-766-3p expression levels and inhibiting activation of S1PR3 through the AKT/mTOR pathway in psoriasis. Our findings help to better understand the pathogenesis of psoriasis and may provide molecular bases for the treatment of psoriasis.

## Figures and Tables

**Table 1:** Sequences of Primers for the qRT-PCR.

	Forward(5' →3')	Reverse(5' →3')
LncRNA H19	ACGTGACAAGCAGGACATGA	TAAGGTGTTCAGGAAGGCCG
S1PR3	TGGTCCCCACTCTTCATCCT	CAGCCAACACGATGAACCAC
GAPDH	GCACCGTCAAGGCTGAGAAC	TGGTGAAGACGCCAGTGGA

## Data Availability

The data used to support the findings of this study are available from the corresponding author upon request.
